# Functional xenobiotic metabolism and efflux transporters in trout hepatocyte spheroid cultures

**DOI:** 10.1039/c4tx00160e

**Published:** 2015-01-29

**Authors:** Chibuzor Uchea, Stewart F. Owen, J. Kevin Chipman

**Affiliations:** a University of Birmingham , School of Biosciences , Birmingham , B15 2TT , UK; b AstraZeneca , Alderley Park , Macclesfield , Cheshire , SK10 4TF , UK . Email: Stewart.Owen@AstraZeneca.com

## Abstract

Prediction of xenobiotic fate in fish is important for the regulatory assessment of chemicals under current legislation. Trout hepatocyte spheroids are a promising *in vitro* model for this assessment. In this investigation, the gene expression and function for xenobiotic metabolism and cellular efflux were characterised. Using fluorescence, transport and real time PCR analysis, the expression and functionality of a variety of genes related to xenobiotic metabolism and drug efflux were assessed in a range of trout hepatocyte culture preparations. Significantly greater levels of expression of genes involved in xenobiotic metabolism and efflux were measured in spheroids (which have been shown to remain viable in excess of 30 days), compared to hepatocytes cultured using conventional suspension and monolayer culture techniques. A transient decline in the expression of genes related to both xenobiotic metabolism and transport was determined during spheroid development, with a subsequent recovery in older spheroids. The most mature spheroids also exhibited an expression profile most comparable to that reported *in vivo*. Functionality of efflux transporters in spheroids was also demonstrated using fluorescent markers and specific inhibitors. In conclusion, the more physiologically relevant architecture in spheroid cultures provides a high functional integrity in relation to xenobiotic metabolism and efflux. Together with the enhanced gene expression and longevity of the model, hepatocytes in spheroid culture may prove to be an accurate alternative model to study the mechanisms of these processes in fish liver and provide an assay to determine the bioaccumulation potential of environmental contaminants.

## Abstract 2

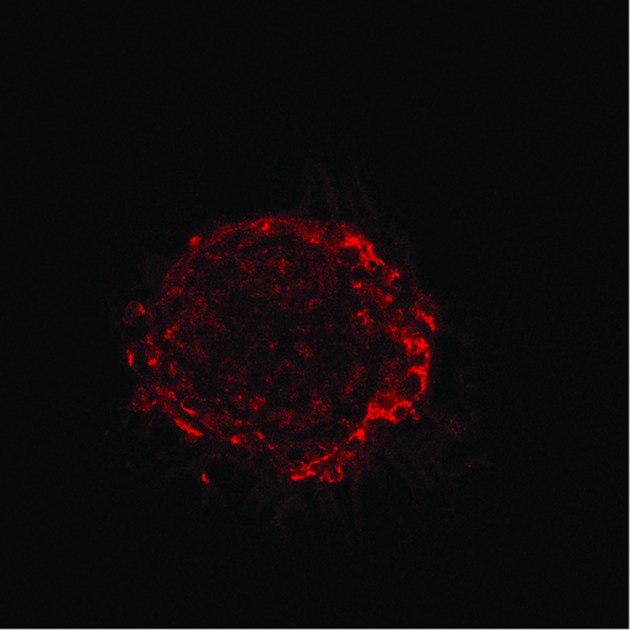
Prediction of xenobiotic fate in fish is important for the regulatory assessment of chemicals under current legislation.

## Introduction

Recently, there has been a significant focus on the benefits of 3D cell cultures, shown to provide an *in vitro* model system circumventing the major limitations (short lived nature and loss of differentiation with time) of traditional monolayer primary cell cultures, maintaining cellular specificity and homeostasis.^1^–^5^ The environment created within 3D cultures involving hepatocytes appears more representative of that in which cells exist in native liver. Hepatocytes are surrounded by other interacting cells, maintaining cell shape and polarity. Together with the development of a more elaborate extracellular matrix, these are features necessary for many specialised functions and potentially responsible for the conserved differentiation status and longevity in spheroids.^6^–^8^ The maintenance of polarity in hepatocyte spheroid culture is also thought to have a key role in the re-formation of bile canaliculi in the system.^6^ Vast differences in the cellular responses of hepatocyte spheroids to chemical exposure have also been described compared to those of conventional cultures. For example, higher resistance to anticancer drugs in 3D tumour cell spheroids in comparison to the same cells in 2D culture has been reported.^9^ The toxic potential of cadmium and silver nanoparticles has also been reported to be significantly reduced in spheroid culture of HepG2 cells when compared to data obtained from conventional cell culture.^10^ These cell responses in spheroid culture are thought to be more reflective of those exhibited *in vivo*, enhancing the predictive power of *in vitro* toxicity testing and use of the system in drug screening has been promoted.^3^,^11^,^12^ Indeed, spheroids are now routinely used as *in vitro* models in cancer and pharmaceutical testing.^9^

Based on their enhanced cytochrome P450 (CYP) activity towards ethoxyresorufin, the potential for 3D spheroid aggregates of trout hepatocytes to be used as a superior *in vitro* alternative to currently used subcellular fractions and monolayer cultures in studies of the metabolism and bioaccumulation of environmental compounds in aquatic organisms has recently been demonstrated.^13^ Alongside these findings, trout hepatocyte spheroids have been shown to outperform 2D cultures biochemically, with significantly enhanced glucose production and albumin synthesis and reduced lactate dehydrogenase leakage.^14^ These studies have also reported the potential benefits of extended longevity (spheroids have been maintained viable and active for over 30 days in our laboratories) conferred by 3D cultures in chronic exposure assessments.

The maintenance of drug metabolism capabilities in spheroids is likely due to well retained gene expression and studies using human hepatocyte spheroid cultures have demonstrated the stable expression of membrane transporters and enzymes related to drug metabolism.^15^ This may arise from the enhanced inter-cellular connectivity of cells and the extended stabilisation period afforded to hepatocytes in this culture form, during which, metabolic activity, levels of gene expression and other features influencing cellular phenotype can be recovered following the cell isolation process.^5^,^16^ Rat hepatocyte spheroids have been reported to undergo an initial period of biochemical and functional turbulence as they mature in early culture and after about 6 days, functional status is said to recover and stabilise.^17^

Another advantage of the use of hepatocytes in spheroid culture for the assessment of bioaccumulation of xenobiotics is the potential incorporation of measurements of transporter function in these preparations. As with the enhanced metabolic performance of hepatocyte spheroids,^13^ we hypothesised that the functional activity of efflux transporters may also be superior in 3D cultures. Proteins of the ATP binding cassette (ABC) facilitate the excretory function in organisms, transporting exogenous and endogenous compounds and/or their metabolites out of cells.^18^ ABC proteins are heavily expressed in the liver, which is a major site for compound elimination.

In recent years, greater recognition of the role of hepatic transporters on the disposition and elimination of compounds, and how these features combine with the metabolic aspects of hepatic clearance, has led to the development of effective methods to accurately assess substrate specificity and affinity for efflux transporters.^19^,^20^ Conventional measurements of hepatic clearance using subcellular fractions, as conducted in environmental bioaccumulation assessment, must assume the cellular uptake of compounds and efficient efflux activity of hepatic transporters. These processes which directly affect bioaccumulation are compound- and species-specific and cannot be assessed using subcellular fractions alone.

Enhanced xenobiotic capabilities have been reported in hepatocyte spheroids isolated from mammalian species. Maintenance of ethoxyresorufin-O-deethylation (EROD) activity and the expression of a range of genes important to hepatocyte function in rat hepatocyte spheroids have been demonstrated.^21^,^22^ An enhanced level of UDP-glucuronosyltransferase (UGT), CYP1A2, CYP2E1 and CYP3A4 activities, as well as Multidrug resistance associated protein 2 (MRP2) expression in a range of hepatocyte cell lines cultured as spheroids compared to those of cells in 2D culture have also been measured.^23^–^25^ Not only are these activities greater in comparison to conventional cultures, but they are also maintained for extensive periods with viability in spheroid culture.^4^

In contrast, very few studies on the use of fish hepatocytes in spheroid culture exist in the literature.^14^,^26^,^27^ The limited information on trout hepatocytes in spheroid culture includes evidence that vitellogenin mRNA expression and secretion is maintained for up to a month in culture and the system has been shown to be responsive to the effects of classical modulators of gene expression.^26^

In fish, as in mammals, proteins of the ABC superfamily play a critical role in the transport of compounds and metabolites into bile.^28^–^30^ This process results in the reduced intracellular concentration and lower toxic potential of compounds, as well as a reduced potential for bioaccumulation.^31^,^32^ ABC transporters are membrane bound proteins consisting of two transmembrane domains, which confer substrate specificity and form the transmembrane channel, and two nucleotide binding domains, which bind and hydrolyse ATP, resulting in the active transport of a wide range of substrates from the cytoplasm and out of cells.^33^–^35^ These proteins are highly conserved and are expressed in a wide range of cell types of all known existing species, suggesting similar, fundamental physiological roles.^36^,^37^ However, despite the physiological importance of the process in fish, relatively little is known about the function and expression of efflux transporters in comparison to their mammalian counterparts,^38^ ABC transporter subtypes are highly expressed in tissues that make up internal and external body boundaries and those involved in excretion, such as liver, intestine and kidney tissue.^38^ These proteins are termed multi drug resistance proteins (MDR) for their role in protecting tumour cells from a wide range of structurally unrelated chemotherapeutics.^18^,^39^,^40^ The diversity of the substrates that proteins of the ABC transporter subfamily can export from cells (including metabolic products of xenobiotic compounds, as well as the parent and some endogenous compounds) is a key feature of their biological importance.

Human ABC proteins are separated into seven subfamilies (A–G) based on their sequence homology.^41^,^42^ In fish, an additional subfamily (H), with one member has also been identified.^34^ The most toxicologically relevant transporters in mammalian species have been identified as the multidrug resistance protein (MDR1, ABCB1), bile salt export pump (BSEP, ABCB11), multidrug resistance-associated proteins 1–3 (MRP1-3, ABCC1-3) and breast cancer resistance protein (BCRP, ABCG2).^19^,^43^ Recent publications have shown that a variety of toxicologically relevant ABC efflux transporters are expressed in trout, with a wide tissue distribution pattern.^29^,^34^,^44^ MDR1, MRP2, BSEP and BCRP have been identified as the efflux transporters with the highest levels of expression in trout liver.^29^,^34^,^44^–^46^

These transporters can be susceptible to inhibition by a number of compounds, affecting their ability to eliminate xenobiotics and resulting in the intracellular accumulation of compounds and possible toxic effects. This is a key issue in aquatic toxicology as a wide range of environmental contaminants are specific inhibitors of ABC transporters and so can increase the sensitivity of organisms to further chemical insult; especially important in the aquatic environment due to the effects of the vast mixtures of chemicals to which organisms are exposed.^29^,^47^–^50^ These interactions will also affect the bioaccumulation potential of compounds, due to potential reductions in clearance efficiency. Despite this knowledge, there is very little information on the retention of such transporter systems in fish hepatocyte spheroids and indeed little in spheroids of any species.

Here we test the hypothesis that drug efflux transporters were functionally active and expressed to a greater extent in trout hepatocyte spheroids when compared to conventional suspension and monolayer cultures and related to expression in whole liver tissue. This may support the inclusion of assessments of compound elimination from hepatocyte spheroids, providing a more accurate comparison to *in vivo* hepatic clearance and enhancing the utilisation of *in vitro* alternatives in chemical safety assessment. To enable their use in such assessments, the system must also be metabolically competent. Therefore the possibility that enhanced xenobiotic metabolism is related to enhanced expression of relevant genes associated with maintenance of the differentiation status in spheroids was tested. The expression of genes related to xenobiotic metabolism and transport was measured during spheroid maturation and compared to freshly isolated hepatocytes and in monolayer culture.

## Materials and methods

### Fish and maintenance

Female, diploid immature rainbow trout (*Oncorhynchus mykiss*), with a wet weight range between 300 g–500 g were held for a minimum 15 day acclimation period at the University of Birmingham prior to first use and fed floating proprietary pellets (GP Pellets, Cheshire, UK), daily. These stock fish were held with permission from the UK Home Office under the Animals (Scientific Procedures) Act 1986 and therefore under authorisation by the University Ethics Committee. The aquaculture conditions were 15 ± 1 °C; 12L:12D photoperiod; non re-circulated water at pH 7.5, hardness >150 mg l^–1^ CaCO_3_ and >80% oxygen saturation. Trout were fasted for 24 hours prior to use. Fish were euthanised following the Schedule I protocol of the Animals (Scientific Procedures) Act 1986 – rendered unconscious by a sharp blow to the head, with subsequent destruction of the brain *via* pithing; following which they were dissected to allow access to the liver. This study complied with regulatory and ethical standards in the UK and the global ethical standards required by the industrial partner AstraZeneca.

### Isolation and culture of hepatocytes

Hepatocytes were isolated using a modified version of the collagenase perfusion technique^51^ in which trout livers were perfused *in situ* through the hepatic portal vein at a flow rate of 2 ml min^–1^, firstly with calcium-free Hanks’ balanced salt solution (Sigma-Aldrich, Poole, UK) for approximately 20 minutes and then with a dissociating solution containing 6.7 mM CaCl_2_, 3.15 mM KCl, 0.3 mM Na_2_HPO_4_, 11.76 mM HEPES, 160 mM NaCl and 260 mg l^–1^ collagenase D (Roche Applied Science, 11088858001) for approximately 15 minutes. A final perfusion with Dulbecco's Modified Eagle's Medium/nutrient mixture F-12 Ham supplemented with l-glutamine, 15 mM HEPES (DMEM [Sigma-Aldrich]) was then provided for approximately 5 minutes to clear the collagenase from the liver. All solutions were pre-incubated at 15 °C. Once digested, livers were removed and cells were isolated from the organ mechanically into DMEM and passed through a 100 μm nylon cell strainer (BD falcon, Massachusetts, USA). Cells were washed 3 times in DMEM following 3 periods of centrifugation (30*g*, 5 minutes, 15 °C) and viability (consistently ≥95%) was measured based on exclusion of 0.04% trypan blue. Isolated primary hepatocytes were plated at a density of 1 × 10^6^ cells cm^–2^ on 24-well collagen type I coated microplates (Iwaki, Japan) and stored in a 15 °C stationary incubator. Hepatocytes in suspension were purified to 1 × 10^6^ cells ml^–1^ in centrifuge tubes, transferred to 50 ml conical flasks and kept in a 15 °C shaking incubator at 90 rpm (Infors-HT, Bottmingen, Switzerland).

### Hepatocyte spheroid culture

Hepatocyte spheroids were prepared using a method previously described in our laboratory which yielded viable spheroids that were able to be maintained in excess of 30 days.^13^ Cells isolated following hepatocyte purification were re-suspended in DMEM which was additionally supplemented with serum replacement 3 (20 ml L^–1^) (Sigma-Aldrich) and an antibiotic/antimycotic solution (10 ml L^–1^) (Sigma-Aldrich, MFCD00130520) at a concentration of 5 × 10^6^ cells ml^–1^. Cell suspensions (5 ml) were plated in sterile, non-treated 50 × 18 mm petri dishes (Sterilin, Newport, UK) and placed on a gyratory shaker (Stuart Scientific, Stone, UK) to aggregate at 50 rpm and 17 °C ± 1 °C. Cell culture medium was changed every 2 days and spheroids were used for gene expression analysis at a range of time points. During spheroid formation, hepatocytes merge together, forming loose cellular aggregates which later develop into larger, variable, asymmetrical clusters. Between days 5–7 post isolation, structures became more uniform in shape, with larger diameters and by day 8, little to no change in their morphological arrangements when compared to earlier time points is evident. At this stage, spheroids in this study exhibited a more homogeneous shape with individual cells no longer clearly visible; a feature considered as a marker of morphological maturity. In this study, day 10 was selected as the first time-point of maturity, to ensure that the morphological maturation process was complete. A number of reports in the literature suggest the optimal diameter for mammalian spheroids in the region of 100–150 μm to allow effective diffusion of oxygen, ensuring that the inner core environment does not become hypoxic.^9^,^52^,^53^ Size analysis of spheroid preparations using flow cytometry showed that the spheroids used in this study ranged from 90–110 μm).

### RNA extraction

Whole liver sections (100 mg) excised during the hepatocyte isolation process, freshly isolated hepatocytes, hepatocytes in monolayer culture and spheroids at a range of time points were collected, washed with PBS (cells) and stored in RNA later (Sigma-Aldrich) at –20 °C.

Total RNA was extracted from stored samples using the trizol, chloroform, glycogen extraction method. Briefly, RNA later was completely removed from samples and whole tissues or cells were homogenised in trizol solution (1 ml, Sigma-Aldrich) in RNase free microcentrifuge tubes (Axygen, California, USA). Following a 5 minute incubation at room temperature, chloroform (200 μl, Sigma-Aldrich) was added and after vigorous shaking, the mixture was centrifuged (12 000*g*, 15 minutes, 4 °C). The upper aqueous phase (500 μl) of the bi-phasic solution was transferred to a fresh microcentrifuge tube and glycogen (10 μg, Fermentas, UK) was added. The aqueous phase was mixed with 70% isopropanol (500 μl, Sigma-Aldrich), incubated at room temperature for 10 minutes and centrifuged (12 000*g*, 10 minutes, 4 °C). The resulting pellet was washed with 75% ethanol (Fisher Scientific, Loughborough, UK), air dried for 10 minutes, re-suspended in RNase-free water (50 μl, Qiagen, Crawley, UK) and incubated at 60 °C for 15 minutes. RNA quality was assessed by agarose gel electrophoresis and quantified by spectrophotometry using a Nanodrop ND1000 (Thermo Scientific, LabTech, East Sussex, UK) and DNA contamination was removed by treatment with a genomic DNA-free treatment kit (Ambion, Austin, U.S.A.). Storage of RNA was at –80 °C.

### cDNA synthesis

Total RNA was used for first-strand cDNA synthesis using the SuperScript II Reverse Transcriptase kit (Invitrogen, Paisley, U.K.) in a thermocycler (Eppendorf Mastercycler Gradient; Eppendorf, Cambridge, UK). The cDNA produced was quantified by spectrophotometry and stored at –20 °C.

### Polymerase chain reaction (PCR)

cDNA was used as a template for PCR and used to validate the sequence-specific primers (Alta Bioscience, Birmingham, U.K) designed for target genes using Primer 3 software or as described in the literature (see Table 1). Stock solutions (10 μM) of each primer were prepared and 50 μl reactions made using components of the DreamTaq DNA Polymerase PCR kit (Fermentas, U.K.), in microcentrifuge tubes. DNA polymerase (1.25 units), forward and reverse primers (10 pmol each), 2 mM dNTP mix (0.2 mM each), 10× DreamTaq buffer (5 μl), template DNA (100 ng) and dH_2_O were combined to a final volume of 50 μl. The PCR programme consisted of an initial 5 minute denaturation period at 95 °C, 35 cycles of denaturation at 95 °C for 1 minute, 1 minute annealing periods at the required primer annealing temperature and extension at 72 °C for 1 minute, followed by a final extension step at 72 °C for 5 minutes in a thermocycler. The resulting products were analysed by DNA gel electrophoresis and comparisons of fragment size were made using a 100 base pair ladder (New England Biolabs, Ipswich, UK).

**Table 1:** PCR primers and product sizes

Gene	Primer sequence	Product size (bp)	Primer concentration (pM)	Calculated average primer efficiency (%)	References
CYP1A	**F**: 5′-GAT GTC AGT GGC AGC TTT GA-3′	104	2.0	101.6	N/A
	**R**: 5′-TCC TGG TCA TCA TGG CTG TA-3′				

CYP2K1	**F:** 5′-CTC ACA CCA CCA GCC GAG AT-3′	164	1.5	97.9	78
	**R**: 5′-CTT GAC AAA TCC TCC CTG CTC AT-3′				

CYP2M1	**F:** 5′-GCT GTA TAT CAC ACT CAC CTG CTT TG-3′	195	1.5	97.2	78
	**R:** 5′-CCC CTA AGT GCT TTG CAT GTA TAG AT-3′				

CYP3A27	**F:** 5′-GAC GGT GGA GAT CAA CG-3′	240	1.0	96.2	79
	**R:** 5′-GAG GAT CTC GAC CAT GG-3′				

UGT	**F:** 5′-ATA AGG ACC GTC CCA TCG AG-3′	113	3.0	97.3	N/A
	**R:** 5′-ATC CAG TTG AGG TCG TGA GC-3′				

MDR1	**F:** 5′-GGA ACT GTC CTC ACC GTG TT-3′	136	3.0	100.5	N/A
	**R:** 5′-GGG GTT TAT TGT CGG TGA TG-3′				

MRP2	**F:** 5′-CCA TTC TGT TCG CTG TCT CA-3′	150	2.0	95.1	N/A
	**R:** 5′-CTC GTA GCA GGG TCT GGA AG-3′				

BCRP	**F:** 5′-AGG CCT GCT GGT GAA CCT G-3′	101	4.0	95.8	46
	**R:** 5′-ACT CAT TAA TTT GGA GAG CTG TTA GTC C-3′				

BSEP	**F:** 5′-CCG ACC AGG GCA AAG TGA TT-3′	101	1.5	98.6	N/A
	**R:** 5′-CAG AAT GGG CTC CTG GGA TAC-3′				

18S rRNA	**F:** 5-TGG AGC CTG CGG CTT AAT TT-3′	170	2.0	97.4	46
	**R:** 5′-ATG CCG GAG TTT CGT TCG TT-3′				

### DNA sample purification and sequencing

PCR products were purified using QIAquick spin columns (Qiagen Ltd, West Sussex, UK), according to the manufacturer's protocol. Following elution, DNA was quantified by spectrophotometry.

Purified DNA samples were then sequenced using an ABI3730 DNA analyser (Applied Biosystems, UK) by the Functional Genomics and Proteomics Unit, University of Birmingham, Birmingham, UK. The sequencing results of the amplified DNA fragments obtained were used in BLAST searches (www.ncbi.nlm.nih.gov/blast) to confirm that the primers amplified the selected genes of interest.

### Real-time PCR

Real-time PCR was conducted using an ABI Prism 7000 Sequence Detection System (Applied Biosystems, USA). cDNA prepared from RNA extracted from various tissue and cell samples were used as the template for reactions with the SensiFast SYBR Hi-Rox kit (Bioline, UK). Five biological replicates were used for each of the target genes, with each individual assessed in triplicate. Samples were run in 96 well plates, each sample containing cDNA (90 ng), 2× sensiFast SYBR (10 μl) using forward and reverse primers at various concentrations (a range of 1–5 pM) dependant on primer efficiency values calculated during optimisation runs, and nuclease free water in a final volume of 20 μl. PCR cycle parameters were: 95 °C, 30 seconds for denaturation; and 60 °C, 30 seconds for combined annealing and extension. No-template controls were also run using sterile dH_2_O.

Melt curves for all samples were plotted and analysed using the ABI Prism 7000 SDS software to ensure only a single product was amplified and primer dimers were not formed. PCR primer efficiencies were calculated using absolute fluorescence values measured in each well and the LinRegPCR software as described by ref. 54, as there can be a significant effect on fold difference calculations using *C*_t_ values calculated from primers with unequal PCR efficiencies. Threshold cycle (*C*_t_) values were recorded for each sample in the linear phase of amplification. Differences in *C*_t_ values were assessed by one way ANOVA (SPSS v16.0).

### Assays of uptake and elimination of fluorescent probes

To assess the role of transporters through the use of inhibitors, the accumulation of fluorescent compounds known to be substrates of particular drug efflux transporters (see Table 2) was assessed following pre-incubations with specific transporter inhibitors. Substrates and inhibitors were dissolved in various solvents as noted and appropriate vehicle controls were used for each. Hepatocyte spheroids were washed twice in PBS and re-suspended in PBS. Inhibitors at a range of concentrations (detailed in Table 3) were added to cultures and incubated for 10 minutes on a gyratory shaker at 50 rpm, following which, the appropriate volume of fluorescent substrate was added to reach the desired concentration. Total solvent concentrations did not exceed 0.5% (v/v) and appropriate solvent controls were always used. Spheroids were incubated in the dark, at 17 °C, on a gyratory platform at 50 rpm, for 60 minutes. After incubation, cells were kept on ice, washed three times in PBS, re-suspended and lysed in 0.1% Triton X-100 in PBS using an ultrasonic water bath (Camlab Transsonic T460, Cambridge, UK) in the dark. Lysate was loaded onto fluorescence 96 well plates (BD Falcon) and fluorescence measured at the relevant wavelength for each substrate (Table 2) using a Bio-Tek FL600 fluorescence plate reader. Fluorescent dye accumulation was measured and compared to control cells exposed to the relevant fluorescent substrate in the absence of the specific inhibitor.

**Table 2:** Fluorescent compounds used in spheroid uptake and elimination studies

Fluorescent compound	Efflux transporter	Concentration (μM)	Solvent (maximum concentration)	Fluorescence wavelengths
Calcein AM	MRP2	10	DMSO (0.4%)	*λ*_ex_: 494 nm; *λ*_em_ 517 nm
Cholyl-lysyl-fluorescein	BSEP	5	Sterile dH_2_O	*λ*_ex_: 485 nm; *λ*_em_ 520 nm
2′,7′-Dichlorodihydrofluorescein diacetate	BSEP	10	DMSO (0.05%)	*λ*_ex_: 495 nm; *λ*_em_ 530 nm
Hoechst 33342	BCRP	10	Sterile dH_2_O	*λ*_ex_: 340 nm; *λ*_em_ 510 nm
Rhodamine 123	MDR1	10	DMSO (0.05%)	*λ*_ex_: 511 nm; *λ*_em_ 534 nm

**Table 3:** Specific inhibitors used in efflux transporter inhibition studies

Efflux transporter	Inhibitor	Concentration range (μM)	Solvent (maximum concentration)
MDR1	Cyclosporine A	0.01–20	DMSO (0.1%)
MRP2	Probenecid	0.5–1000	NaOH (0.01%)
BCRP	Sodium taurocholate	1–1500	Sterile dH_2_O
BSEP	Ko 143	0.01–20	DMSO (0.4%)

### Confocal microscopy

Cells exposed to various fluorescent compounds were washed in PBS and re-suspended in phenol red free PBS. Suspended cells were pipetted into glass bottom culture dishes (MatTek Corporation, Massachusetts, USA) for imaging. Images were acquired using a Leica TCS SP2 confocal microscope (Leica Microsystems, Milton Keynes, UK) with a 63× oil immersion objective lens. Fluorochromes were excited using an argon laser at the wavelengths described previously.

### Statistical analysis

Statistical analysis was conducted using SPSS version 16.0. Tests for normality and homogeneity of variance were conducted using the Shapiro Wilk and Levene's tests respectively. Data meeting the assumption criteria for parametric tests were analysed using the Independent samples *T*-test and data not meeting these criteria were analysed using the Kruskal-Wallis and Mann-Whitney *U* test (non parametric). *P* values <0.05 were deemed significant.

## Results

Investigations were conducted on hepatic preparations derived from the same subset of fish. Gene expression analyses were conducted on cells isolated from the same five individual fish and functional transporter assays using specific inhibition were conducted using three individual fish. The confocal images provided are representative.

To provide a temporal overview of the changes in gene expression during the various stages in spheroid development, expression data is presented in the form of heat maps displaying the range of expression levels of each gene, recorded in each of the types of hepatocyte preparation. Values recorded are cycle threshold (*C*_t_) values, which display an inverse relationship to the amount of nucleic acid measured in samples, representing absolute expression. Therefore, the lowest extreme *C*_t_ values, represent the highest level of expression. The scale bars above the maps indicate the range of recorded *C*_t_ values (from lowest to highest).

### Status of efflux transporter gene expression in hepatocyte preparations

**Fig. 1:**
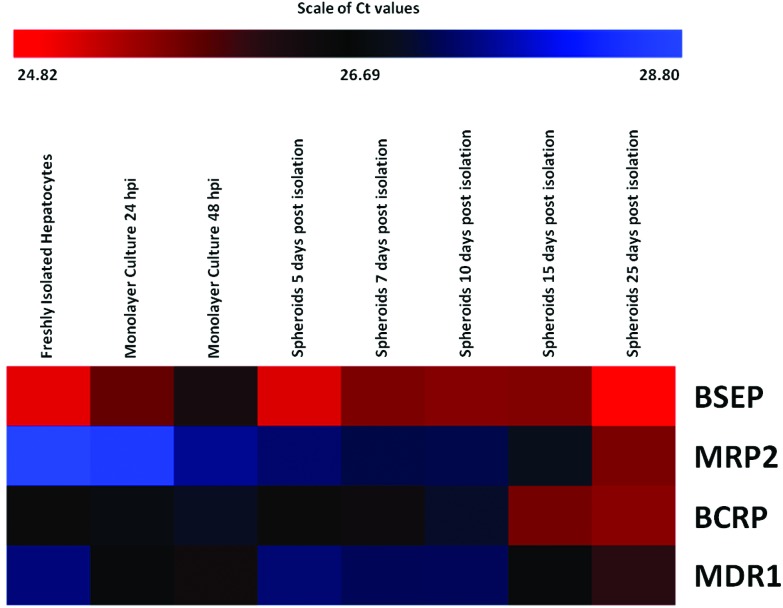
Expression levels of genes related to ABC efflux transporters in freshly isolated hepatocytes, hepatocytes in monolayer culture (24 and 48 hours post isolation) and hepatocyte spheroids. The bar above the heat map indicates the scale of *C*_t_ values measured in cell samples with red representing the lowest extreme *C*_t_ value, therefore the greatest level of expression; blue representing the highest extreme *C*_t_ value, therefore the lowest level of expression; and black representing the median level of expression (*n* = 5 fish).

Differences in transcript levels of the transporters of interest (MDR1, BSEP, MRP2 and BCRP) were seen in the different hepatocyte cultures assessed (freshly isolated suspensions, monolayer cultures 24 and 48 hours post isolation and spheroid cultures 5, 7, 10, 15 and 25 days post isolation) (Fig. 1). Expression levels of BSEP and BCRP in monolayer culture were lower than those measured in freshly isolated hepatocytes and, following a recovery in early spheroid culture, an increase in expression was evident in mature spheroids.

In conventional monolayer culture, there was a decline in the expression of both BSEP and BCRP at 24 and 48 hours post isolation, when compared to the level measured in freshly isolated hepatocytes. This was seen by an increase in Ct value and a corresponding fold change of 0.55 ± 0.18 (*p* < 0.05, Mann-Whitney *U* test) and 0.63 ± 0.13 (*p* < 0.05, Mann-Whitney *U* test) for BSEP and BCRP respectively in monolayer culture 24 hours post isolation and 0.51 ± 0.20 (*p* < 0.05, Mann-Whitney *U* test) and 0.79 ± 0.13 (*p* > 0.05, Mann-Whitney *U* test) respectively in monolayer culture 48 hours post isolation (Fig. 1). In contrast to these declines, increases in the expression of MRP2 and MDR1, relative to levels measured in freshly isolated hepatocytes, were measured in monolayer cultures at the same time points. MRP2 expression in hepatocytes 48 hours post isolation exhibited a significant fold change of 2.07 ± 0.44 (*p* < 0.05, Mann-Whitney *U* test) in comparison to freshly isolated hepatocytes and there were also significant fold changes of 1.52 ± 0.25 and 2.09 ± 0.49 (*p* < 0.05, Mann-Whitney *U* test) in the expression of MDR1 in monolayer cultures 24 hours and 48 hours post isolation respectively, when compared to freshly isolated hepatocytes (Fig. 1).

Efflux transporter expression levels measured in hepatocytes in spheroid culture were greater than, or equal to, levels measured in conventional primary hepatocyte cultures. During the development of spheroid structures (between 5–10 days post isolation), expression of genes for efflux transporters remained relatively stable (with the exception of BCRP expression in spheroids 10 days post isolation) although, gradual increases in the expression of MRP2 and MDR1 were seen. This increase in MRP2 expression in early spheroids, continued at subsequent time points. The highest levels of expression of all efflux transporters were measured in mature spheroids, with significant increases in the expression of MRP2 and MDR1 (7.88 ± 1.02 and 2.59 ± 0.28 fold respectively) recorded in spheroids 25 days post isolation.

In all hepatocyte preparations and at all time points, BSEP was the efflux transporter exhibiting the greatest level of expression (Fig. 1). This observation was in accordance with the expression of BSEP in whole liver samples taken from the same individual fish and in accord with published data.^34^ In contrast, MRP2 exhibited the lowest level of expression in freshly isolated hepatocytes and hepatocytes in monolayer culture (comparable to the expression in whole liver) and the level of MDR1 expression was lowest in hepatocyte spheroids compared to other preparations at all time points. The pattern of expression of the transporters of interest was constant at all stages of spheroid culture.

### Functional activity of efflux transporters in spheroid hepatocytes

**Fig. 2:**
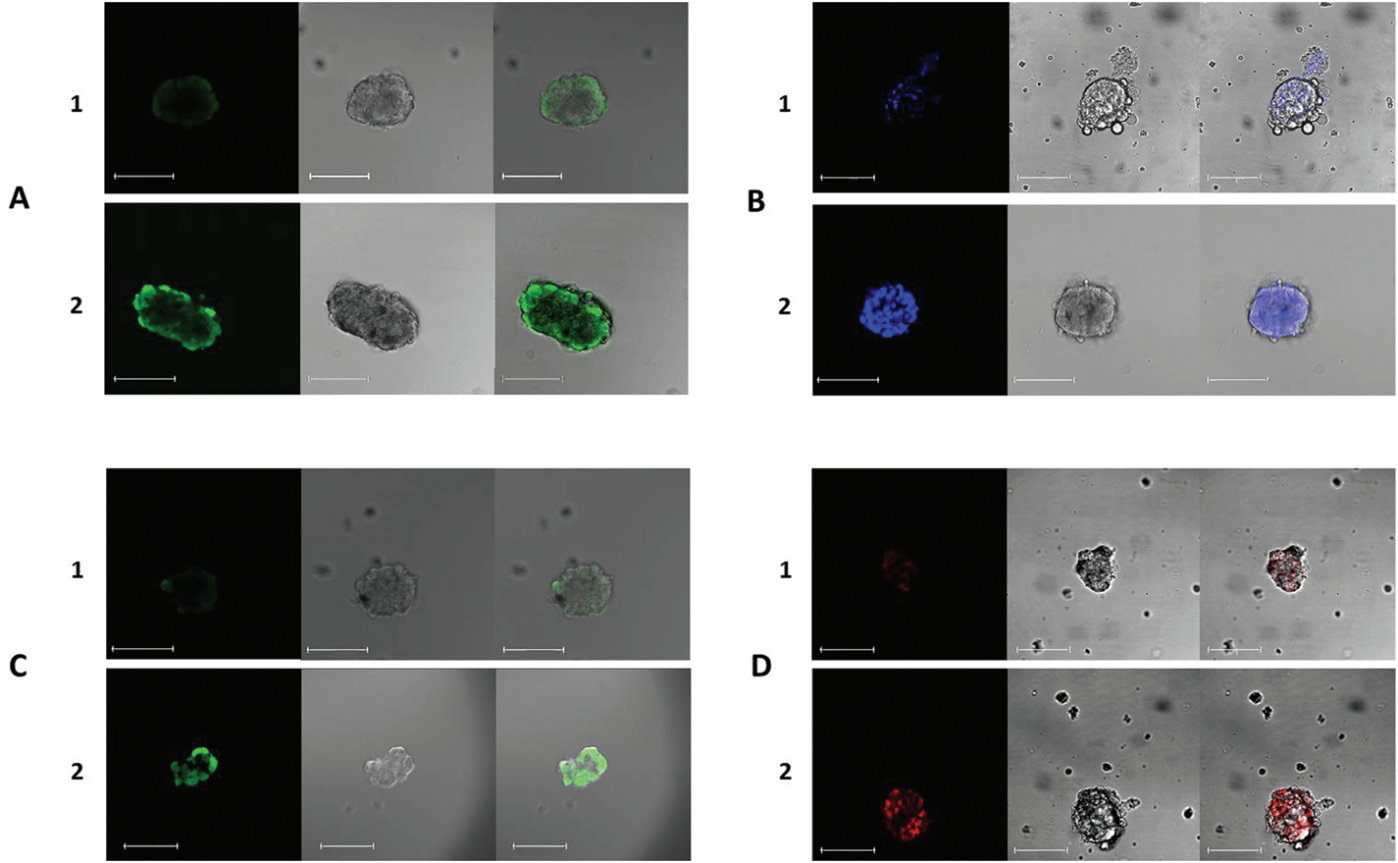
Representative visualisation of the accumulation of DHFDA (A), H33342 (B), calcein-AM (C) and rhodamine 123 (D) in hepatocytes in spheroid cultures. A–D 1 show representative controls and A–D 2 show representative spheroids following treatment with sodium taurocholate (A), Ko143 (B), probenecid (C) and cyclosporin A (D). The intracellular accumulation of fluorescent probes in spheroids was observed using confocal microscopy. At the maximum concentrations of inhibitors used, a greater accumulation of fluorescence was observed in spheroids, in comparison to untreated cells in each case. Spheroids used from 15 days post isolation. Images from left to right: fluorescence channel; Bright field scan; merged image. All scale bars indicate 100 μm.

The functions of the efflux transporters of interest were assessed using fluorescent substrates and inhibitors successfully employed in other studies (*e.g.*ref. 29, 32, 46, 49 and 55). The fluorescent substrates used were calcein-acetoxymethylester (Ca-AM), rhodamine 123 (Rh123), hoechst 33342 (H33342) and 2′,7′-dichlorodihydrofluorescein diacetate (DHFDA). The intracellular presence and accumulation of these compounds in spheroids was observed and confirmed by confocal microscopy (Fig. 2). Increases in intracellular accumulation of substrates as a result of specific inhibition were quantified using a fluorescence plate reader and compared to the fluorescence measurements recorded in control cells. All inhibitors investigated caused significant changes in substrate accumulation.

**Fig. 3:**
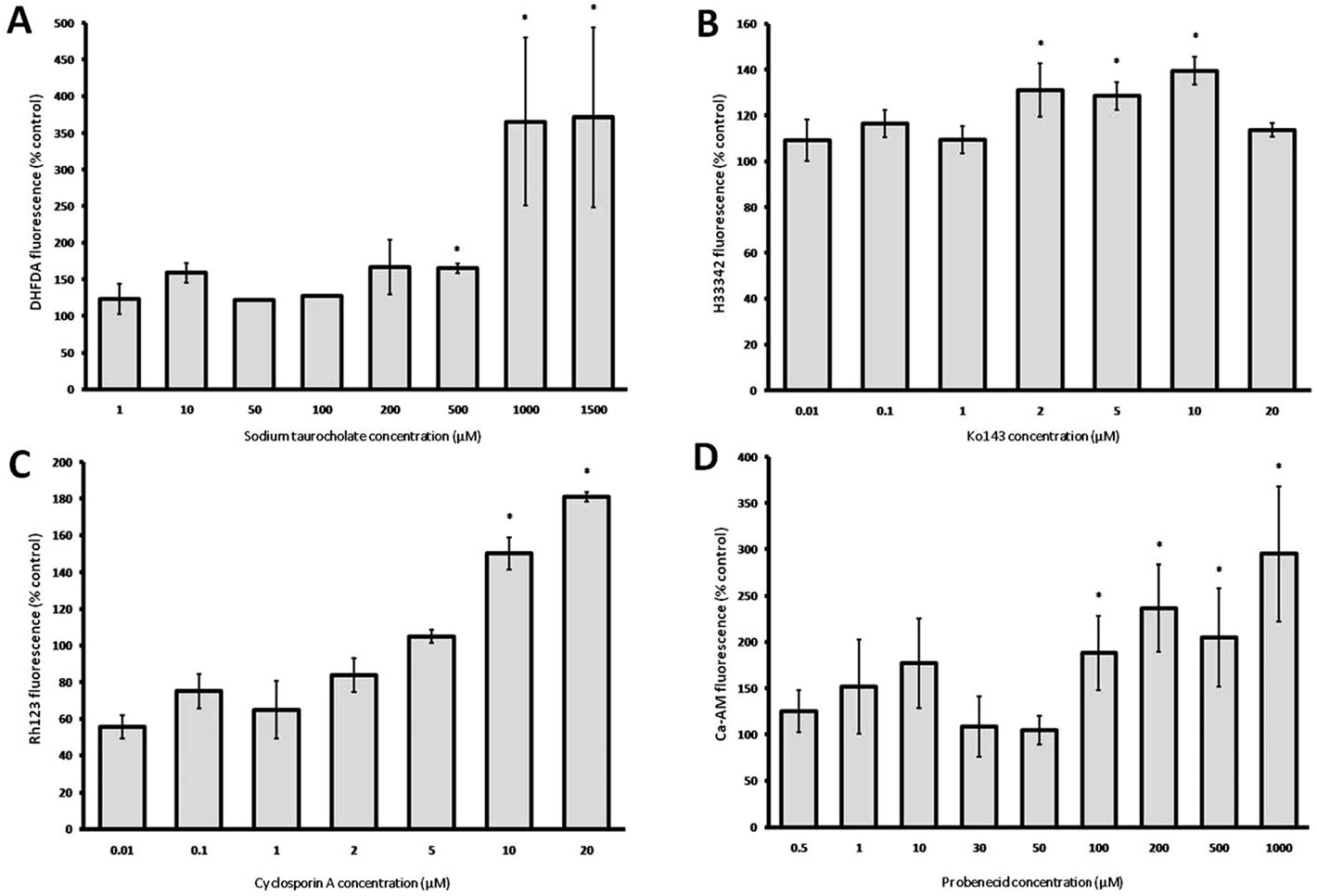
The effect of sodium taurocholate (A), Ko143 (B), probenecid (C) and cyclosporin A (D) on the accumulation of the substrates shown in each case within hepatocyte spheroid cultures. Data represent mean fluorescence accumulation relative to uninhibited controls and expressed as a percentage increase ±SEM. Control fluorescence accumulation = 100%; *p < 0.05 (significantly greater than control), Mann-Whitney *U* test (*n* = 3 fish).

The highest maximal fluorescence accumulation in spheroids was seen with sodium taurocholate, used to inhibit BSEP. A 3.70 fold increase in DHFDA accumulation was measured at 1.0 and 1.5 mM concentrations of sodium taurocholate. Inhibition of DHFDA efflux was not significant below concentrations of 500 μM (Fig. 3A). In comparison, fluorescence accumulation as a result of BCRP inhibition by Ko143 was considerably lower, with a maximum 1.38 fold increase in H33342 accumulation. BCRP inhibition by Ko143 caused significant increases in fluorescence at concentrations of 2 μM and above (*p* < 0.05, Mann-Whitney *U* test) however, a distinct concentration-dependent increase of H33342 was not evident as a result of BCRP inhibition (Fig. 3B).

In contrast, inhibition of MDR1 activity was seen at cyclosporin A concentrations of 10 and 20 μM with concentration-dependent increase in intracellular rhodamine 123 accumulation of 1.5 fold and 1.8 fold respectively (Fig. 3C). Probenecid produced concentration-dependent increases of calcein-AM accumulation, through the inhibition of MRP2 in spheroids (Fig. 3D). A significant increase in fluorescence accumulation (*p* < 0.05, individual samples *T* test) was measured in cells exposed to concentrations of 100 μM and above, with a maximum 2.95 fold accumulation measured at 1 mM.

### Re-formation of bile canaliculi in spheroid hepatocytes

**Fig. 4:**
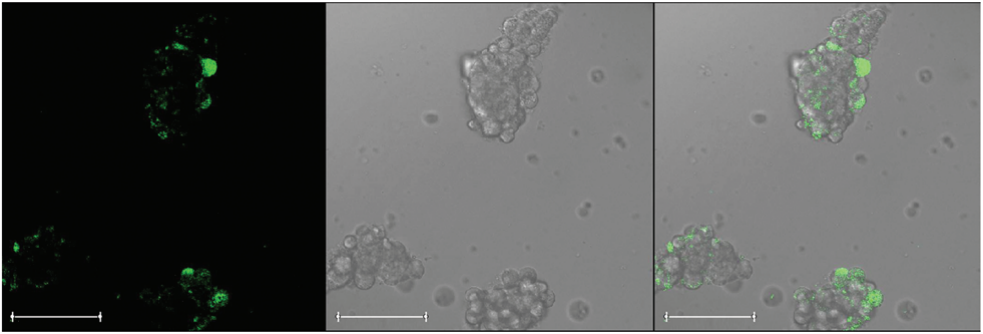
Confocal imaging of cholyl-lysyl-fluorescein (CLF) distribution within spheroids. Spheroids were treated with CLF for 30 minutes and images show evidence of the concentration of the fluorescent compound in these structures. Staining was punctate and did not correspond to a pan-cellular localisation within individual cells. This may reflect the presence, in part, of canalicular structures between cells from which bile release from hepatocyte spheroids is mediated. Spheroids used from 15 days post isolation. Images from left to right: fluorescence channel; Bright field scan; merged image. All scale bars indicate 100 μm.

The fluorescent bile acid cholyl-lysyl-fluorescein (CLF) was found to be taken up into spheroids and subsequently released with time. Spheroids were treated with fluorescent CLF and allowed to efflux the marker over a period of 30 minutes, revealing some evidence of the concentration of pools of punctate fluorescence, which did not correspond to the structure of whole cells (Fig. 4). It is possible that this punctate labelling is reflective of the presence of canalicular structures from which bile release from spheroids is mediated.

### Changes in the expression of genes involved in xenobiotic metabolism in different hepatocyte preparations

**Fig. 5:**
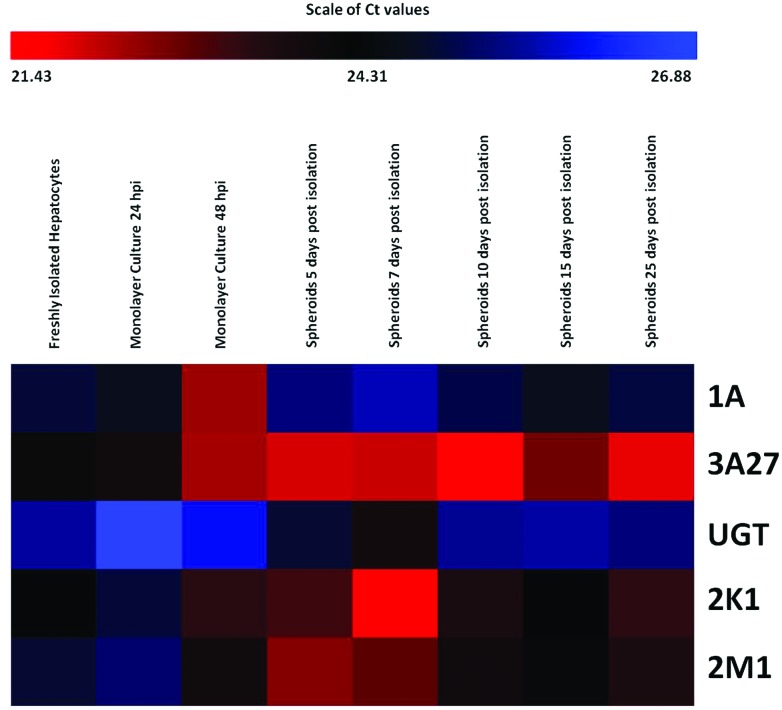
Relative expression levels of genes related to xenobiotic metabolism in freshly isolated hepatocytes, hepatocytes in monolayer culture (24 and 48 hours post isolation) and hepatocyte spheroids. The bar above the heat map indicates the scale of *C*_t_ values measured in cell samples with red representing the lowest extreme *C*_t_ value, therefore the greatest level of expression; blue representing the highest extreme *C*_t_ value, therefore the lowest level of expression; and black representing the median level of expression.

The transcript levels of CYP1A, CYP2K1, CYP2M1, CYP3A27 and UGT were also assessed and compared in the same hepatocyte preparations as used in transporter assessments. A number of gene specific changes in expression levels in different hepatocyte preparations were identified (Fig. 5). A decline in the expression of UGT, CYP2K1 and CYP2M1 was evident in early monolayer culture, followed by a recovery in late monolayer culture and early stages of spheroid culture. Expression levels of CYP1A and CYP3A27 gradually increased in this timeframe; with a sharp decline in CYP1A expression evident in spheroids 5 days post isolation. Levels of expression of the genes of interest appeared to be relatively more stable in more mature spheroids (Fig. 5).

With the exception of CYP1A and CYP3A27, the expression levels of all genes in monolayer culture 24 hours post isolation were less than, or equal to, those measured in freshly isolated hepatocytes. However, in monolayer cultures 48 hours post isolation, expression increased to a level greater than that measured in freshly isolated hepatocytes (with the exception of UGT expression). In the majority of cases, expression levels measured in hepatocyte spheroids were greater than measured in freshly isolated hepatocytes and hepatocytes in monolayer culture. The expression of CYP1A was the exception to this, with the level of expression lower at all stages of spheroid maturation, than measured in monolayer culture and less than, or equal to, the level recorded in freshly isolated hepatocytes. Experience in our laboratory has also demonstrated difficulty in replicating *in vivo* activity of CYP1A (*via* EROD) using primary monolayer cultures.^56^

In spheroid culture, expression levels of genes related to xenobiotic metabolism were generally highest at 5 and 7 days post isolation, with a small decline at 10 and 15 days post isolation; with the exception of CYP1A and CYP3A27 at 10 days post isolation and CYP1A in spheroids 15 days post isolation. However, expression levels remained greater than that measured in freshly isolated hepatocytes. Gene expression later increased at 25 days post isolation (with the exception of CYP1A), to levels greater than measured at 10 days post isolation and less than but closer in comparison to those detected at 5 and 7 days post isolation. Mature spheroids (10, 15 and 25 days post isolation) appeared to retain the most similar pattern of expression in comparison to that observed in the whole liver (Fig. 5). However, caution must be exercised when making such comparisons as a smaller proportion of hepatocytes (in the region of 80%), contribute to the total liver protein in whole liver.

### Principal component analyses

**Fig. 6:**
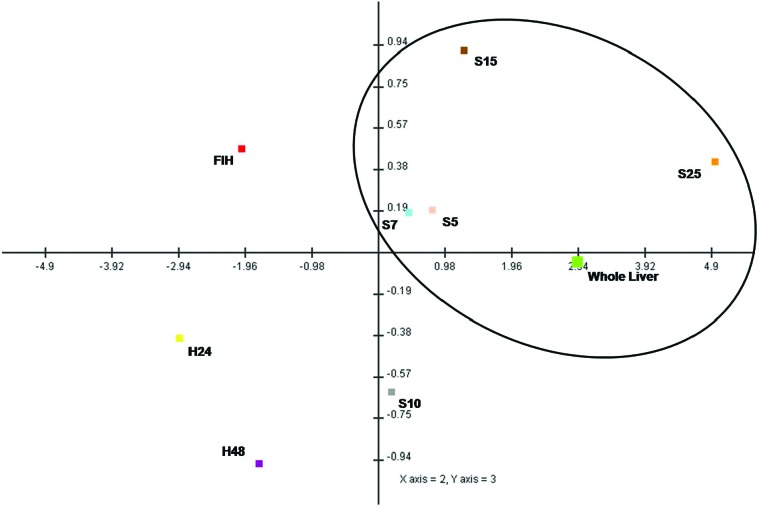
Principal components analysis (PCA) scores plot for the profile of expression of genes related to xenobiotic efflux in whole liver and all hepatocyte preparations. The plot shows separation of *in vivo* and *in vitro* preparations along the PC2 and PC3 axes. There was a clear separation of hepatocytes in monolayer culture at the greatest extreme (bottom left quadrant) from the whole liver sample. In comparison, the expression profile of freshly isolated hepatocytes was closer to that of the whole liver than to the other conventional cell culture types. Variability existed in the expression profile of spheroid hepatocytes according to stage of culture, however, spheroids at all time points displayed a better resemblance to the expression profile of the whole liver. Spheroid hepatocytes 5, 7 and 25 days post isolation showed the best similarity to the expression profile *in vivo*. FIH: freshly isolated hepatocytes; H24: monolayer culture 24 hpi; H48: monolayer culture 48 hpi; S5: spheroid culture 5 dpi; S7: spheroid culture 7 dpi; S10: spheroid culture 10 dpi; S15: spheroid culture 15 dpi; S25: spheroid culture 25 dpi.

The principal component analysis plot (Fig. 6) demonstrates the overall predominant similarity of spheroids to whole liver with respect to drug efflux transporters.

## Discussion

The transport of compounds through cell membranes is an important feature of xenobiotic detoxification, having a key influence on the absorption, distribution, elimination, toxicity and efficacy of compounds. For the majority of compounds, transport and metabolism must be assessed together to allow accurate predictions of *in vivo* disposition, physiological effects and xenobiotic fate to be made.^2^,^57^ Importantly, this feature is lacking in assays currently used for the assessment of the bioaccumulation potential of environmental contaminants, conducted using subcellular fractions, where the cellular uptake and elimination of a xenobiotic is assumed.^58^ The aim of the present investigation was to explore the potential advantages of the use of hepatocyte spheroids (pertaining to the greater expression and activity of drug transporters), making such assays more informative, with respect to the evaluation of bioaccumulation potential and therefore more relevant to the detoxification of compounds *in vivo*.

In this study, the expression and activity of all of the efflux transporters of interest was identified in hepatocyte spheroids at different stages of maturity. Although some variation in expression levels was evident at different time points, transcript levels were generally greater than those measured in conventional cultures. A general down-regulation of expression in monolayer culture was evident, most likely to be associated with cellular de-differentiation in these cultures. Following a recovery period in early spheroid culture, an increase in the expression of genes related to xenobiotic metabolism and transport was evident in mature spheroids, reaching the highest values in aggregates 25 days post isolation.

The 3D structure of hepatocytes, with enhanced cell–cell and cell-extracellular matrix interactions is thought to be responsible for the restoration and maintenance of cellular polarisation and differentiation, lost during the isolation process and in conventional culture.^17^,^59^ De-differentiation and the ensuing loss of gene expression and specific cellular functions has traditionally been problematic in conventional cultures.^2^ Cellular differentiation is known to be mediated through extracellular signals *via* locally acting molecules within the extracellular matrix and from adjacent cells. These interactions directly affect cell function and behaviour, regulated at gene expression level.^60^ The importance of differentiation status on the expression of the uptake transporter organic anion transporter polypeptide (OATP) and the efflux transporter MRP1 has previously been shown in trout cell lines.^34^,^61^ Therefore, maintenance of cellular differentiation as seen in spheroid hepatocytes, confers significant advantages, making this model an attractive alternative for *in vitro* studies of drug transporters.

In common with expression in whole liver samples, in all *in vitro* preparations and at all time points in culture, the expression of BSEP was the greatest of all transporters investigated. This is in accordance with a study in which BSEP expression in trout liver tissue was measured as 750 fold and 114 fold greater than MDR1 and MRP2 respectively.^29^ High hepatic BSEP expression is also a common feature measured in mammalian systems, however in contrast, expression of the transporter in a study using a variety of trout cell lines (including 3 of hepatic origin) was among the lowest of those investigated.^46^ In addition, no functional activity of BSEP or BCRP was identified in trout hepatocyte-derived cell lines, despite being the highest expressed transporters *in vivo*. This is a key issue for these cell lines and has significant implications for using that model in examining xenobiotic metabolism and transport. BSEP is involved in the efflux of a wide range of endogenous and exogenous compounds, with a particular role in the secretion of bile acids into the bile canaliculus.^34^,^62^ The lack of OATP which imports bile acids into hepatocytes and the absence of a canalicular structure has been suggested as a cause for the low expression and lack of BSEP function in trout cell lines.^46^,^63^ Rat hepatocyte couplets exhibit bile canalicular structures into which bile constituents are secreted^64^,^65^ indicating the importance of cell–cell structural interactions. In the current investigation, it was observed that hepatocytes connected *via* spheroid structure retain BSEP expression and function and there is some evidence of canalicular structure (Fig. 4), similar to structures observed in rat hepatocyte spheroids.^6^ The uptake and release of CLF, occurred in trout spheroids where release was inhibited by sodium taurocholate. The concentration of punctate fluorescent staining which did not correspond to the structure of whole cells suggested the presence of functional canalicular structures (Fig. 4). These findings again present advantageous features of spheroids for use in studies of xenobiotic transport compared to the use of hepatocyte derived cell lines lacking such structures.

Similar to the expression of genes related to transport, expression of genes related to xenobiotic metabolism were generally greater in spheroids than in freshly isolated hepatocytes or in monolayer culture. With the exception of CYP1A, expression levels in spheroids did not fall below those measured in other hepatocyte preparations.

Temporal differences were evident in the profile of expression of all genes (both metabolism and transport related) in hepatocyte spheroids, with cells 10 days post isolation showing the greatest deviation with respect to the *in vivo* profile and spheroids 25 days post isolation demonstrating the greatest analogy. In this study, the point at which little to no change in microscopic structural arrangements were seen was used as the indicator of morphological maturation. At this stage, spheroids exhibited a more homogeneous shape than those of earlier time points. It may be possible that this marker of morphological maturation does not necessarily match a similar period of gene expression, where levels are expected to stabilise. It has been reported that in rat hepatocyte spheroids, biochemical and functional turbulence occurs as spheroids mature in early culture, recovering and stabilising after about 6 days.^17^ The expression profile of spheroids in this study at 10 days post isolation was markedly different from that of other spheroids. The temporal and transient decline in gene expression measured at this stage may be reflective of a similar period of turbulence and instability. Indeed, changes in cellular protein content, increases in levels of albumin synthesis and changes in metabolism, to levels more reflective of those measured in intact tissue has been reported during the transition from immature to mature spheroids.^14^ The exact stage at which gene expression stabilises in trout hepatocyte spheroids may be later than that of the observed morphological maturation, potentially explaining the expression differences observed at 10 days post isolation. It remains unclear however, exactly at which stage trout hepatocytes can be noted as mature and it has been suggested that despite classifying spheroids as mature after a period of 6–8 days, the fusion of cells may continue up to 16 days.^14^ Functional stability may prove to be a better indicator of maturity for use of spheroids in specific assays. Also, xenobiotic metabolism activities in rat hepatocyte spheroids remained significantly higher than in rat hepatocyte monolayer cultures.^4^

It is difficult to directly compare expression levels between isolated hepatocyte preparations and whole liver due to differences in cellular composition. Hepatocytes have been shown to be quantitatively dominant in trout liver, contributing around 80–85% liver volume.^66^,^67^ Whole liver samples include at least 10 other, non-parenchymal cell types^68^ which play a role in the function, differentiation and gene regulation of hepatocytes, through inter-cellular signalling.^69^,^70^ It has been reported that co-culture systems maintaining parenchymal and non-parenchymal cells concurrently, show improved morphological characteristics and increased drug metabolism capabilities^71^–^73^ and this could be applied to trout hepatocyte spheroids in the future. Despite this, hepatocyte spheroids retained a more representative profile of gene expression related to the xenobiotic detoxification process than the monolayer cultures.

To assess functionality of the transporter proteins encoded by the genes of interest in spheroid cultures, transporter activities were investigated using specific fluorescent substrates and inhibitors used in mammalian efflux transporter assays, many of which have been used in previous studies involving fish *in vitro* hepatic preparations.^32^,^46^,^55^ Significant differences in the accumulation of substrates were seen in spheroids at the highest inhibitory concentrations of all compounds used, suggesting that the transporters of interest identified in spheroids were functional and susceptible to inhibition. In common with the high expression of BSEP in spheroids, its inhibition resulted in the greatest substrate accumulation. High levels of DHFDA accumulation in spheroids was in agreement with the inhibition of BSEP in trout hepatocyte monolayer cultures,^29^ highlighting the potential importance of biliary excretion pathways in rainbow trout, as demonstrated by the detection of xenobiotics and metabolites in bile collected from whole fish.^74^

A lack of knowledge of the exact specificities of inhibitors and the broad and overlapping substrate specificities of efflux transporters proves problematic in the accuracy of functional transporter assays. Especially for the assessment of transporters in fish as the assumption of activities is based on the mammalian literature. Recent studies have highlighted the relatedness of MDR1, BSEP and MRP-group transporters, raising questions on the inhibitory specificity of cyclosporin A and the transporter specificities of rhodamine 123 and calcein-Am among others.^29^,^32^,^46^,^75^ An important feature to explore further in hepatocyte spheroids is the use of transporter inhibitor co-exposure, to block multiple efflux pathways, to circumvent the lack of knowledge of transporter substrate specificity. The expression of individual transporters could also be down-regulated using RNAi, however problems with transfection efficiency in primary cells, especially those of piscine origin, have been reported.^76^,^77^ It may also be possible that inhibition of individual transporters can result in the up-regulation of other, closely related transporters, with overlapping specificity. Nevertheless, irrespective of the precise specificity of inhibitors, it is clear that transporter efflux was measurable and susceptible to inhibition (Fig. 3).

A number of environmentally relevant compounds have been shown to modify the expression and function of efflux transporters in aquatic organisms as well as genes involved in xenobiotic metabolism.^20^,^36^,^46^ Both may contribute to the accumulation of compounds in organisms when exposed to mixtures of environmental contaminants.^32^,^47^ As a result, information on the efflux of compounds and their potential to inhibit transporter function should be included in the assessment of environmental contaminants. In this study, emphasis has been placed on the efflux of compounds. However, in the prediction of the potential of a compound to bioaccumulate, the kinetics of uptake, influenced by factors such as lipid solubility and intestinal transporter activity should also be considered in association with xenobiotic metabolic capacity analysis. Information gained experimentally in this area could be used to develop further predictive models that might support the environmental risk assessment of xenobiotics and industrial chemicals.

This study is the first to assess trout ABC transporters in long term culture, showing the improved expression of the key efflux transporters, in comparison to other cell culture models. The spheroidal system has the potential to be adapted for routine use in the assessment of environmental contaminants as this would not only confer advantages of longevity and maintained metabolic activity, but will also enable the incorporation of measurements of efflux and inhibition, affirming such cultures as a much more informative model for the screening of bioaccumulation potential. The system will be of high interest in the context of potential industrial use in a range of *in vitro* metabolic and transport assessments of chemical safety (especially with respect to assessments of chronic exposure) and overcomes the de-differentiation associated with more conventional hepatocyte cultures that has plagued researchers for many years.

Liver tissues used in this study were derived from freshly euthanised rainbow trout supplied from stocks at the University of Birmingham. These stock fish were held with permission from the UK Home Office under the Animals (Scientific Procedures) Act 1986 and therefore under authorisation by the University Ethics Committee. Since they were killed under a schedule 1 method, they did not undergo a scientific procedure by definition of the Act. This study complied with regulatory and ethical standards in the UK and the global ethical standards required by the industrial partner AstraZeneca. One of the principle aims of this work was to establish and characterise an *in vitro* model that will potentially reduce the numbers of fish used in future environmental tests required by regulatory authorities. Currently approximately 30 000 fish are used per year in the UK alone, more in other countries; and these tests are required by regulatory authorities around the world (see ; https://www.gov.uk/government/publications/user-guide-to-home-office-statistics-of-scientific-procedures-on-living-animals). By developing these *in vitro* organoid models, we hope to contribute to reducing these numbers in the future. Furthermore, since the fish used in this study are not exposed to chemicals whilst alive, this represents a significant refinement in the methodology, and likely reduces the effects of endogenous stress responses. This study represents a significant step towards developing future models that will be acceptable to regulatory authorities, and therefore is a key contribution towards our endeavours to further the 3Rs (Reduction, Refinement, Replacement) of vertebrate animals in toxicology.

## Conflict of interest

C. Uchea was in receipt of a scholarship co-funded by the Biotechnology and Biological Sciences Research Council (BBSRC) and AstraZeneca (Safety Health and Environment Research Programme) to J. K. Chipman, an employee of the University of Birmingham. S. F. Owen is an employee of AstraZeneca. AstraZeneca is a bio-pharmaceutical manufacturer that discovers, develops, manufactures and markets a wide range of pharmaceuticals that necessarily require an environmental risk assessment including the potential to bioaccumulate in aquatic species. The authors declare no conflicts of interest.
